# Supplementary material for: Effective Preparation of [18F]Flumazenil Using Copper-Mediated Late-Stage Radiofluorination of a Stannyl Precursor
Source: Molecules. 2022 Sep 13;27(18):5931. doi: 10.3390/molecules27185931 (PMC9505495; doi:10.3390/molecules27185931)
Supplement: Supplementary file 1 [file molecules-27-05931-s001.zip › molecules-1821424-supplementary.pdf]

Effective preparation of $[^{18}F]$Flumazenil using copper mediated late stage radiofluorination of a stannyl precursor

Mohammad B. Haskali [1,2*], Peter D. Roselt [2], Terence J O'Brien[3] , Craig A. Hutton [4,5], Idrish Ali [3] , Lucy Vivash [3], Bianca Jupp [3]

1 Sir Peter MacCallum Department of Oncology, The University of Melbourne, Melbourne, VIC 3010, Australia

2 The Radiopharmaceutical Research Laboratory, The Peter MacCallum Cancer Centre, Melbourne, VIC 3000, Australia; peter.roselt@petermac.org

3 Department of Neuroscience, Central Clinical School, Monash University, Melbourne, VIC 3004, Australia; Terence.OBrien@monash.edu (T.J.O.); idrish.ali@monash.edu (I.A.); lucy.vivash@monash.edu (L.V.); bianca.jupp@monash.edu (B.J.)

4 School of Chemistry, The University of Melbourne, Melbourne, VIC 3010, Australia; chutton@unimelb.edu.au

5 Bio21 Molecular Science and Biotechnology Institute, The University of Melbourne, Melbourne, VIC 3010, Australia

* Correspondence: mo.haskali@petermac.org; Tel.: +61-(3)-8559-6913

^{1}H NMR Spectrum of stannyl-mazenil 3

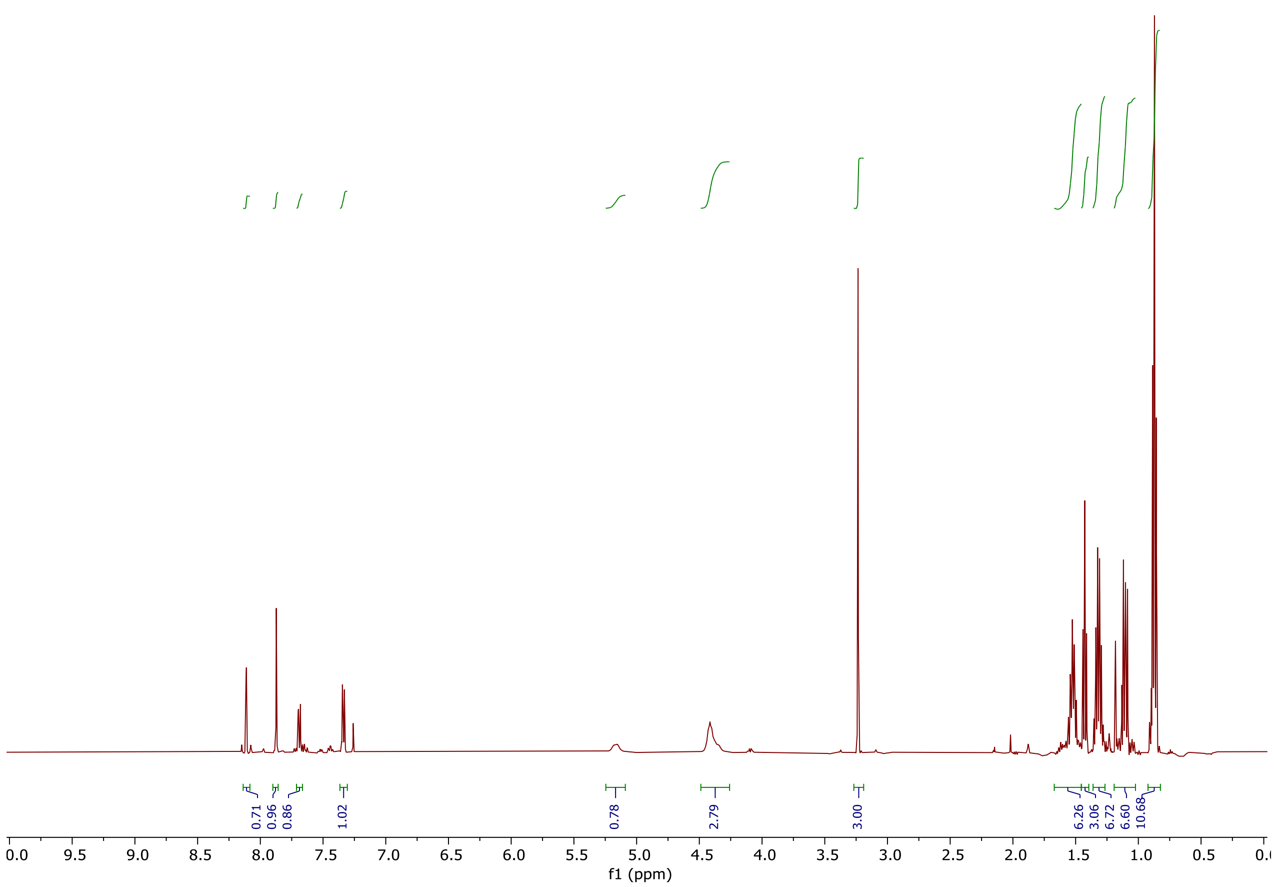

MS Analysis of flumazenil 1

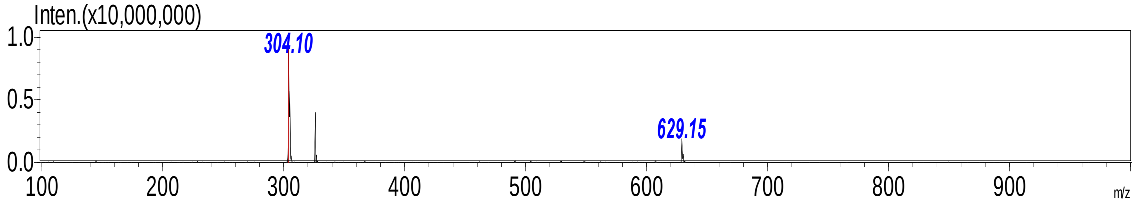

MS Analysis of stannyl-mazenil 3

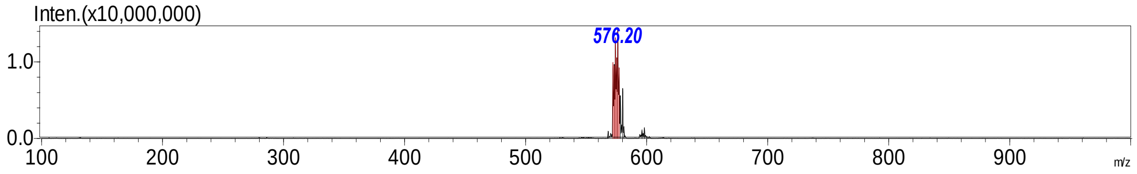

MS Analysis of hydroxy-mazenil 4

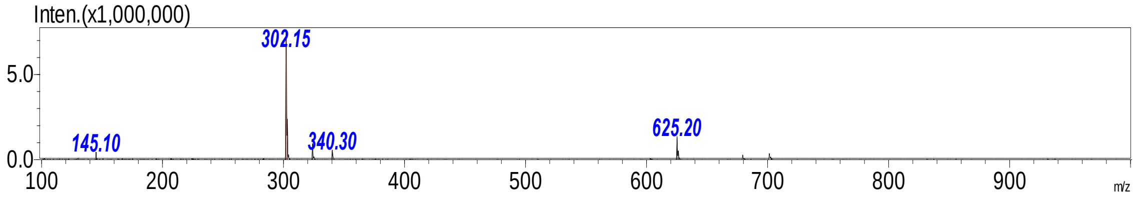

MS Analysis of des-fluoro-flumazenil 5

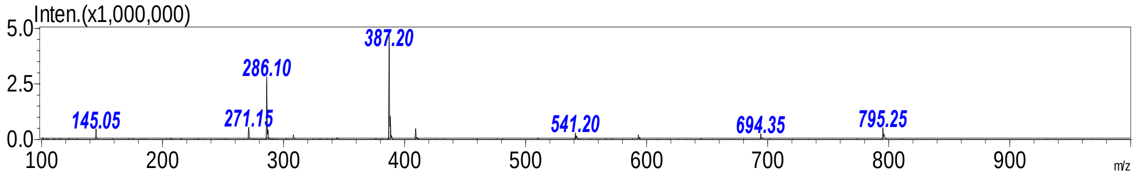

MS Analysis of dimeric-mazenil 6

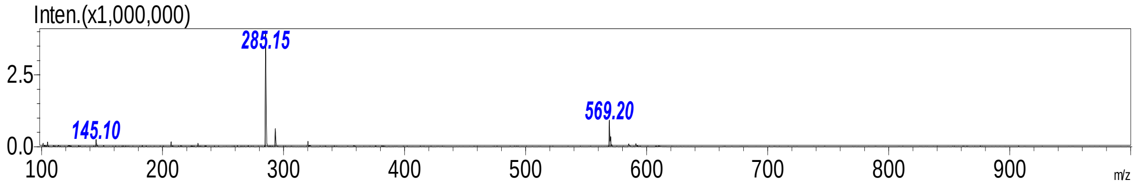

MS-MS of Flumazenil 1; fragmentation of M+H ion at *m/z* 304

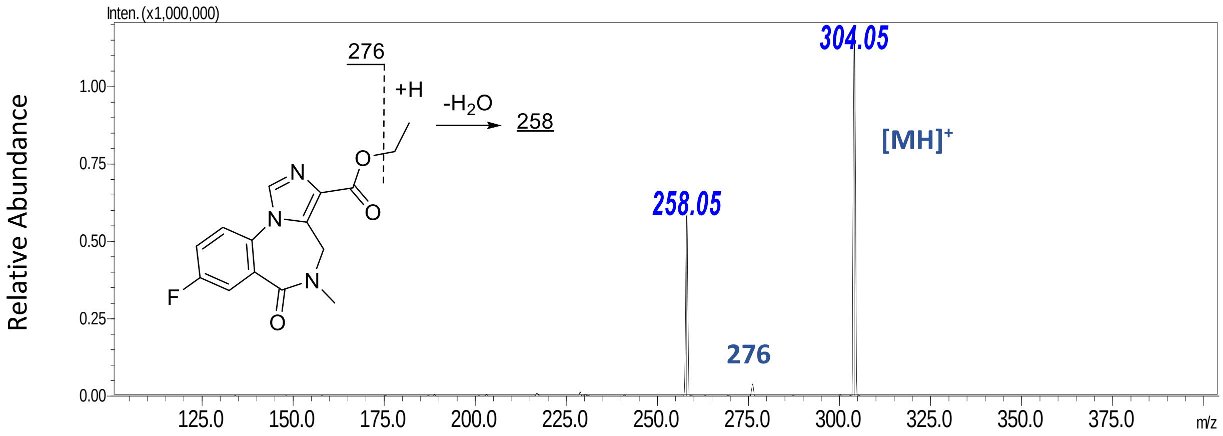

Fragmentation profile at collision energy 10 eV

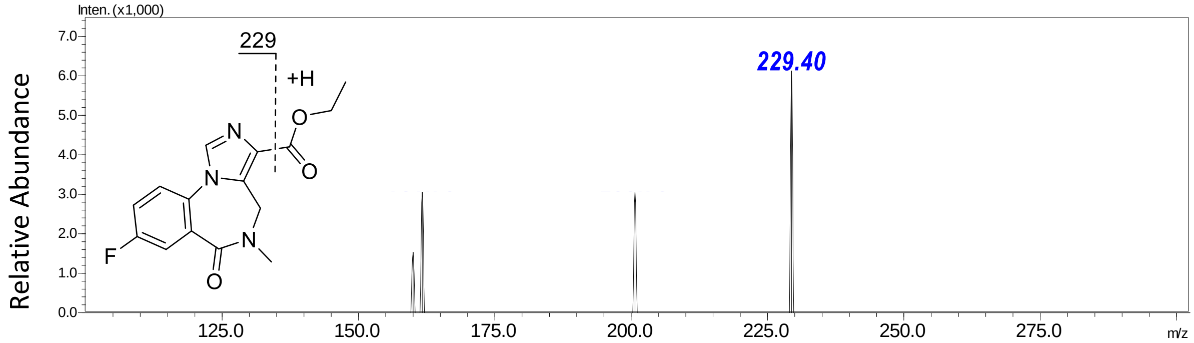

Fragmentation profile at collision energy 35 eV

MS-MS of desfluoro-flumazenil 5; fragmentation of M+H ion at *m/z* 286

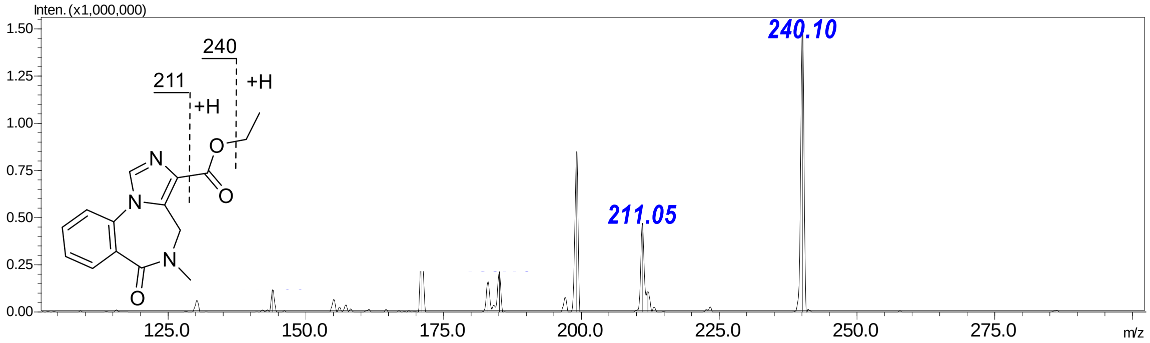

Fragmentation profile at collision energy 10 eV

MS-MS of hydroxyl-mazenil 4; fragmentation of M+H ion at *m/z* 302

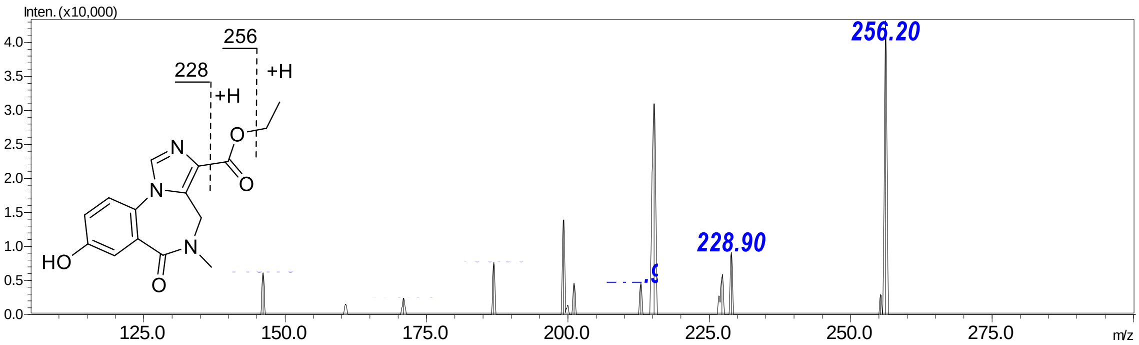

Fragmentation profile at collision energy 10 ev

HPLC chromatogram of purified [^{18}F]FMZ 1

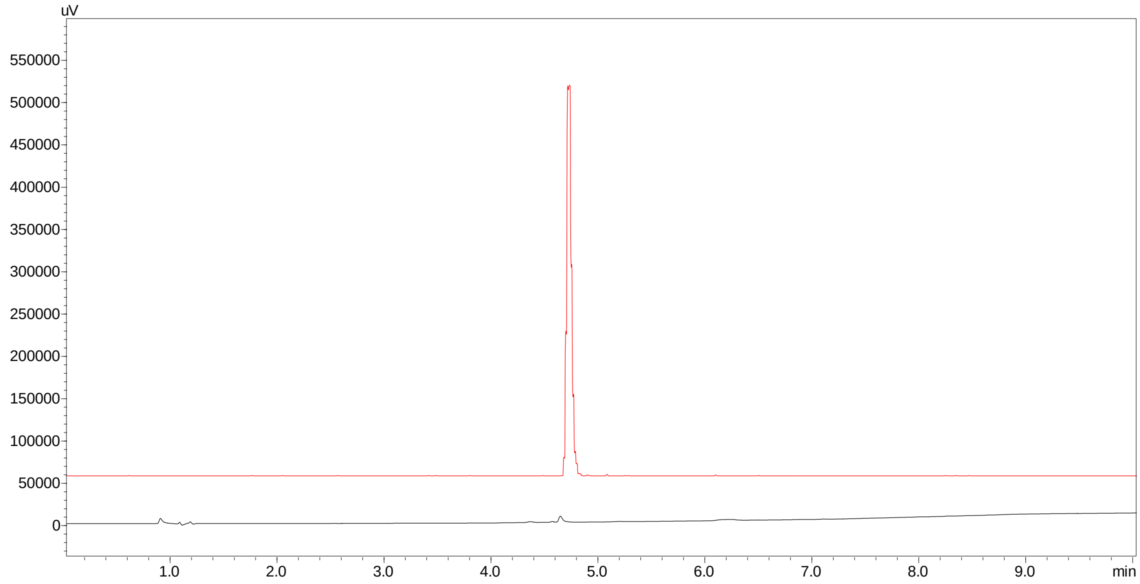

HPLC analysis performed on Kinetex 5 µm XB-C18 4.6 x 150 mm column, 0.1% TFA in 15–90% MeCN:H_2O over 7 min.

[^{18}F]FMZ 1 co-injected with reference standard

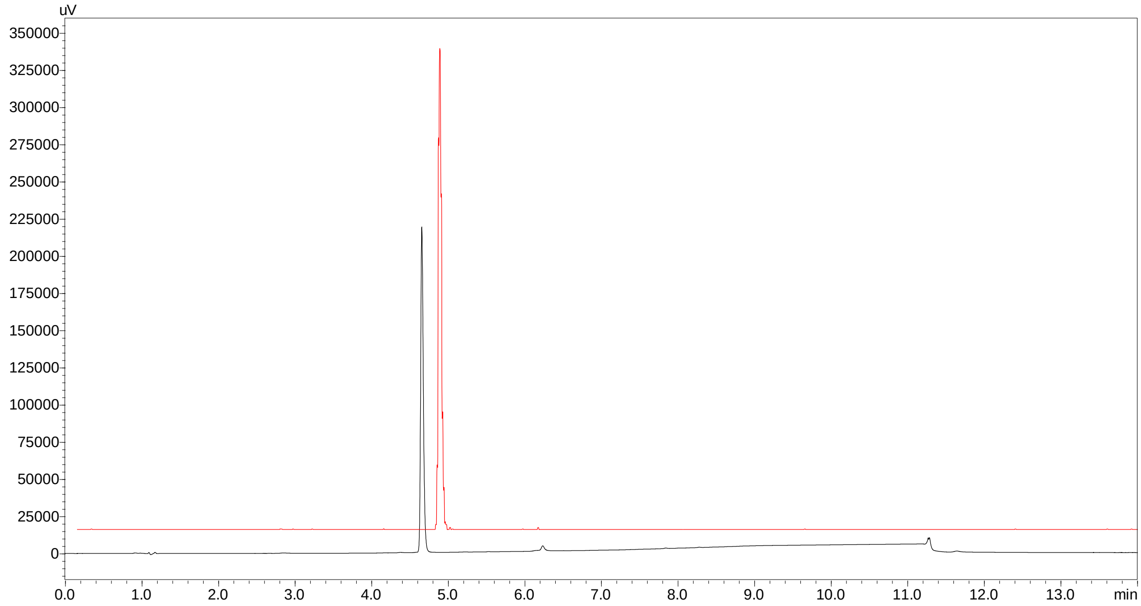

HPLC analysis performed on Kinetex 5 µm XB-C18 4.6 x 150 mm column, 0.1% TFA in 15–90% MeCN:H_2O over 7 min.

FMZ 1 Calibration curve

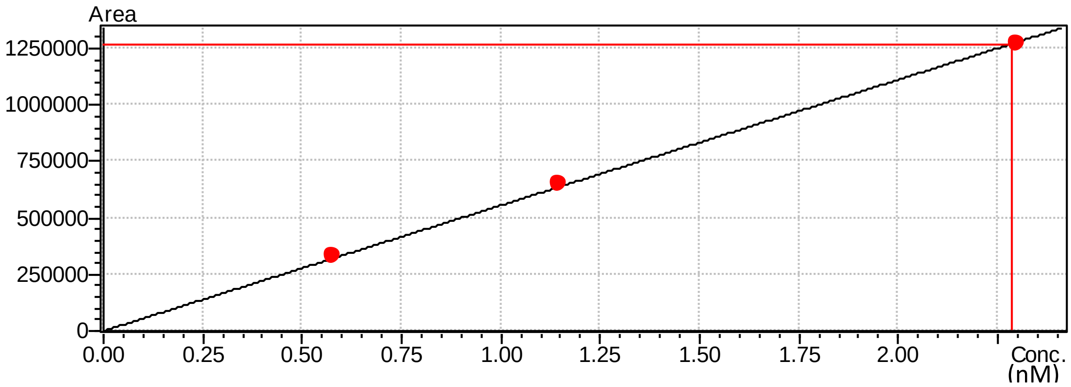

Calibration Information - Compound ID# 1

Compound ID#: 1

Final Column Display: (•) Area/Height ( ) File Name

Curve Fit Type: Linear

R = 0.9999384

R^2 = 0.9998767

RSS = 2.412432e+008

%RSD = 1.492423

Y = (556684)X + (0)

Date Processed: 10/02/2021 1:25:22 PM

OK | Cancel | Help

Level	Cal. Point	Average	# of Reps.	Area 1
1	☑	328255	1	328255
2	☑	651778	1	651778
3	☑	1272810	1	1272810

[^{18}F]FMZ Automated Production Recipe

Step	Step Message	Step Condition	Step Time (seconds)	V01 (0=off, 1=on)	V02 (0=off, 1=on)	V03 (0=off, 1=on)	V04 (0=off, 1=on)	V05 (0=off, 1=on)	V06 (0=off, 1=on)	V07 (0=off, 1=on)	V08 (0=off, 1=on)	V09 (0=off, 1=on)	V10 (0=off, 1=on)	V11 (0=off, 1=on)	V12 (0=off, 1=on)	V13 (0=off, 1=on)	V14 (0=off, 1=on)
1	Load KHCO3(2 mg in 0.1mL Water), MeCN (0.9mL), K222	0	0	0	0	0	0	0	0	0	0	0	0	0	0	0	0
2	Load FMZ precursor and Pyridine/Cu in DMA (1mL) into via	0	0	0	0	0	0	0	0	0	0	0	0	0	0	0	0
3	Load 0.1% TFA in H20:MeCN (60:40, 3mL) into vial 6	0	0	0	0	0	0	0	0	0	0	0	0	0	0	0	0
4	Load Saline (5mL) into vial 10	0	0	0	0	0	0	0	0	0	0	0	0	0	0	0	0
5	Load Ethanol (1mL) into vial 9	0	0	0	0	0	0	0	0	0	0	0	0	0	0	0	0
5	Load saline (10mL) into vial 8	0	0	0	0	0	0	0	0	0	0	0	0	0	0	0	0
7	Load Water (30ml) into HPLC Flask 1	0	0	0	0	0	0	0	0	0	0	0	0	0	0	0	0
8	Pressurize Reactor 1	0	**6**	0	0	0	0	0	0	0	0	0	0	0	0	0	0
9	Waiting for pressure leak in Reactor 1	0	**9**	0	0	0	0	0	0	0	0	0	0	0	0	0	0
10	Pressure testing Reactor 1	**20**	**1**	0	0	0	0	0	0	0	0	0	0	0	0	0	0
11	Install conditined QMA cartridge at position QMA	0	0	0	0	0	0	0	0	0	0	0	0	0	0	0	0
12	Install conditoned C-18 cartridge at position SPE B	0	0	0	0	0	0	0	0	0	0	0	0	0	0	0	0
13	Check HPLC Eluent A = 0.1% TFA in water	0	0	0	0	0	0	0	0	0	0	0	0	0	0	0	0
14	Check HPLC Eluent B = 0.1% TFA in MeCN	0	0	0	0	0	0	0	0	0	0	0	0	0	0	0	0
15	Connect final product collection vial	0	0	0	0	0	0	0	0	0	0	0	0	0	0	0	0
16	Pressurize reagent vials	0	**9**	0	0	0	0	0	0	0	0	0	0	0	0	0	0
17	Waiting for pressure leak in reagent vials	0	**9**	0	0	0	0	0	0	0	0	0	0	0	0	0	0
18	Pressure testing reagent vials	**22**	**1**	0	0	0	0	0	0	0	0	0	0	0	0	0	0
19	Ready for F- from cyclotron - Press NEXT when transfered	**1**	0	0	0	0	0	0	0	0	0	0	0	0	0	0	0
20	Trapping F- onto QMA cartridge	0	**60**	0	0	0	0	0	0	0	0	0	0	0	0	**1**	**1**
21	Elution of QMA cartridge into Reactor 1	0	**60**	**1**	0	0	0	0	0	0	0	0	0	0	0	0	0
22	Recording activity in Reactor for RCY	**5**	**2**	**1**	0	0	0	0	0	0	0	0	0	0	0	0	0
24	MeCN for azeotropic drying	0	**60**	0	**1**	0	0	0	0	0	0	0	0	0	0	0	0
24	Drying fluoride (gas + vacuum)	0	**340**	0	0	0	0	0	0	0	0	0	0	0	0	0	0
25	Drying fluoride (vacuum only)	0	**120**	0	0	0	0	0	0	0	0	0	0	0	0	0	0
26	Cooling Reactor 1	**12**	**400**	0	0	0	0	0	0	0	0	0	0	0	0	0	0
27	Addition of precursor in tBuOH-MeCN (vial 3) to Reactor 1	0	**25**	0	0	0	**1**	0	0	0	0	0	0	0	0	0	0
28	Reaction at 140C for 20min	0	**600**	0	0	0	0	0	0	0	0	0	0	0	0	0	0
29	Cooling Reactor 1	**12**	**400**	0	0	0	0	0	0	0	0	0	0	0	0	0	0
30	Transferring Reactor 1 to Intermediate Vial 1	0	**45**	0	0	0	0	0	0	0	0	0	0	0	0	0	0
31	Addition of 0.1%TFA water (vial 6) to Reactor 1	0	**30**	0	0	0	0	0	**1**	0	0	0	0	0	0	0	0
32	Pressurize gas lines	0	**2**	0	0	0	0	0	0	0	0	0	0	0	0	0	0
33	Transferring Reactor 1 to Intermediate Vial 1	0	**45**	0	0	0	0	0	0	0	0	0	0	0	0	0	0
34	Injecting into HPLC loop - Waiting for fluid detector 1 ON	**30**	**60**	0	0	0	0	0	0	0	0	0	**1**	0	0	0	0
35	Injecting into HPLC loop - Waiting for fluid detector 1 OFF	**32**	**60**	0	0	0	0	0	0	0	0	0	**1**	0	0	0	0
36	HPLC purification of [18F]FBR (40min gradient to 5%A 95%	0	0	0	0	0	0	0	0	0	0	0	0	0	0	0	0
37	Collecting product peak into HPLC Flask 1 - Click NEXT to	0	0	0	0	0	0	0	0	0	0	0	0	0	0	0	0
38	Trapping purified [18F]FBR onto C-18 at SPE-B	0	**200**	0	0	0	0	0	0	0	0	0	0	0	**1**	0	0
39	Washing FMZ with Saline	0	**60**	0	0	0	0	0	0	0	0	0	0	**1**	0	0	0
40	Elution of FMZ into dose vial	0	**40**	0	0	0	0	0	0	0	0	**1**	0	0	0	0	0
41	Dilution with 10 ml saline	0	**100**	0	0	0	0	0	0	0	**1**	0	0	0	0	0	0
42	Recording activity in Final Product Vial for RCY	**6**	**2**	0	0	0	0	0	0	0	**1**	0	0	0	0	0	0
43	Transfer of final product vial out	0	**250**	0	0	0	0	0	0	0	0	0	0	0	0	0	0
44	Stopping HPLC 2	0	**2**	0	0	0	0	0	0	0	0	0	0	0	0	0	0
45	End of synthesis - Click ABORT to finish	**2**	0	0	0	0	0	0	0	0	0	0	0	0	0	0	0

[^{18}F]FMZ Automated Production Recipe Continued

V15 (0=off, 1=on)	V16 (0=off, 1=on)	V17 (0=off, 1=on)	V18 (0=off, 1=on)	V19 (0=off, 1=on)	V20 (0=off, 1=on)	V21 (0=off, 1=on)	V22 (0=off, 1=on)	V23 (0=off, 1=on)	V24 (0=off, 1=on)	V25 (0=off, 1=on)	V26 (0=off, 1=on)	V27 (0=off, 1=on)	V28 (0=off, 1=on)	V29 (0=off, 1=on)	V30 (0=off, 1=on)	V31 (0=off, 1=on)	V32 (0=off, 1=on)	V33 (0=off, 1=on)	V34 (0=off, 1=on)	V35 (0=off, 1=on)	V36 (0=off, 1=on)	V37 (0=off, 1=on)	V38 (0=off, 1=on)	V39 (0=off, 1=on)	V40 (0=off, 1=on)	V41 (0=off, 1=on)	V42 (0=off, 1=on)	V43 (0=off, 1=on)	V44 (0=off, 1=on)	V45 (0=off, 1=on)	V46 (0=off, 1=on)
0	0	0	0	0	0	0	0	0	0	0	0	0	0	0	0	0	0	0	0	0	0	0	0	0	0	0	0	0	0	0	0
0	0	0	0	0	0	0	0	0	0	0	0	0	0	0	0	0	0	0	0	0	0	0	0	0	0	0	0	0	0	0	0
0	0	0	0	0	0	0	0	0	0	0	0	0	0	0	0	0	0	0	0	0	0	0	0	0	0	0	0	0	0	0	0
0	0	0	0	0	0	0	0	0	0	0	0	0	0	0	0	0	0	0	0	0	0	0	0	0	0	0	0	0	0	0	0
0	0	0	0	0	0	0	0	0	0	0	0	0	0	0	0	0	0	0	0	0	0	0	0	0	0	0	0	0	0	0	0
0	0	0	0	0	0	0	0	0	0	0	0	0	0	0	0	0	0	0	0	0	0	0	0	0	0	0	0	0	0	0	0
0	0	0	0	0	0	0	0	0	0	0	0	0	0	0	0	0	0	0	0	0	0	0	0	0	0	0	0	0	0	0	0
0	1	0	0	0	0	0	0	0	0	0	0	0	0	0	0	0	0	0	0	0	0	0	0	0	0	0	0	0	0	0	0
0	0	0	0	0	0	0	0	0	0	0	0	0	0	0	0	0	0	0	0	0	0	0	0	0	0	0	0	0	0	0	0
0	0	0	0	0	0	0	0	0	0	0	0	0	0	0	0	0	0	0	0	0	0	0	0	0	0	0	0	0	0	0	0
0	0	0	0	0	0	0	0	0	0	0	0	0	0	0	0	0	0	0	0	0	0	0	0	0	0	0	0	0	0	0	0
0	0	0	0	0	0	0	0	0	0	0	0	0	0	0	0	0	0	0	0	0	0	0	0	0	0	0	0	0	0	0	0
0	0	0	0	0	0	0	0	0	0	0	0	0	0	0	0	0	0	0	0	0	0	0	0	0	0	0	0	0	0	0	0
0	0	0	0	0	0	0	0	0	0	0	0	0	0	0	0	0	0	0	0	0	0	0	0	0	0	0	0	0	0	0	0
0	0	0	0	0	0	0	0	0	0	0	0	0	0	0	0	0	0	0	0	0	0	0	0	0	0	0	0	0	0	0	0
0	0	0	0	0	0	0	0	0	0	0	0	0	0	0	0	0	0	0	0	0	0	0	0	0	0	0	0	0	0	0	0
0	0	0	0	0	0	0	0	0	0	0	0	0	0	0	0	0	0	0	0	0	0	0	0	0	0	0	0	0	0	0	0
0	0	0	0	0	0	0	0	0	0	0	0	0	0	0	0	0	0	0	0	0	0	0	0	0	0	0	0	0	0	0	0
0	0	0	0	0	0	0	0	0	0	0	0	0	0	0	0	0	0	0	0	0	0	0	0	0	0	0	0	0	0	0	0
0	1	0	0	0	0	1	0	0	0	0	0	0	0	0	0	0	0	0	0	0	0	0	0	0	0	0	0	0	0	0	0
1	0	0	0	0	0	1	0	0	0	0	0	0	0	0	0	0	0	0	0	0	0	0	0	0	0	0	0	0	0	0	0
1	0	0	0	0	0	1	0	0	0	0	0	0	0	0	0	0	0	0	0	0	0	0	0	0	0	0	0	0	0	0	0
0	0	0	0	0	0	1	0	0	0	0	0	0	0	0	0	0	0	0	0	0	0	0	0	0	0	0	0	0	0	0	0
0	1	1	0	0	0	1	0	0	0	0	0	0	0	0	0	0	0	0	0	0	0	0	0	0	0	0	0	0	0	0	0
0	0	1	0	0	0	1	0	0	0	0	0	0	0	0	0	0	0	0	0	0	0	0	0	0	0	0	0	0	0	0	0
0	1	0	0	0	0	1	0	0	0	0	0	0	0	0	0	0	0	0	0	0	0	0	0	0	0	0	0	0	0	0	0
0	0	0	0	0	0	1	0	0	0	0	0	0	0	0	0	0	0	0	0	0	0	0	0	0	0	0	0	0	0	0	0
0	0	0	0	0	0	0	0	0	0	0	0	0	0	0	0	0	0	0	0	0	0	0	0	0	0	0	0	0	0	0	0
0	0	0	0	0	0	0	0	0	0	0	0	0	0	0	0	0	0	0	0	0	0	0	0	0	0	0	0	0	0	0	0
0	1	0	1	1	1	0	0	0	0	0	0	0	0	0	0	0	0	0	0	0	0	0	0	0	0	0	0	0	0	0	0
0	0	0	0	0	0	1	0	0	0	0	0	0	0	0	0	0	0	0	0	0	0	0	0	0	0	0	0	0	0	0	0
0	0	0	0	0	0	0	0	0	0	0	0	0	0	0	0	0	0	0	0	0	0	0	0	0	0	0	0	0	0	0	0
0	1	0	1	1	1	0	0	0	0	0	0	0	0	0	0	0	0	0	0	0	0	0	0	0	0	0	0	0	0	0	0
0	0	0	0	0	0	0	0	1	0	0	0	0	0	0	0	0	0	0	0	0	0	0	0	0	0	0	0	0	0	0	0
0	0	0	0	0	0	0	0	1	0	0	0	0	0	0	0	0	0	0	0	0	0	0	0	0	0	0	0	0	0	0	0
0	0	0	0	0	0	0	0	0	0	0	0	0	0	0	0	0	0	0	0	0	0	0	0	0	0	0	0	0	0	0	0
0	0	0	0	0	0	0	0	0	1	0	0	0	0	0	0	0	0	0	0	0	0	0	0	0	0	0	0	0	0	0	0
0	0	0	0	0	0	0	1	0	0	0	0	0	0	0	0	0	0	0	0	0	0	0	0	0	0	0	0	0	0	0	0
0	0	0	0	0	0	0	0	0	0	0	0	0	0	0	0	0	0	0	0	0	0	0	0	0	0	0	0	0	0	0	0
0	0	0	0	0	0	0	0	0	0	0	0	0	0	0	0	0	0	0	0	0	0	0	0	0	0	0	0	0	0	0	1
0	0	0	0	0	0	0		0	0		0	0	0	0	0	0	0	0	0	0	0	0	0	0		0		0		0	1
0	0	0	0	0	0	0	0	0	0	0	0	0	0	0	0	0	0	0	0	0	0	0	0	0	0	0	0	0	0	0	0
0	0	0	0	0	0	0	0	0	0	0	0	0	0	1	0	0	0	0	0	0	0	0	0	0	0	0	0	0	0	0	0
0	0	0	0	0	0	0	0	0	0	0	0	0	0	0	0	0	0	0	0	0	0	0	0	0	0	0	0	0	0	0	0
0	0	0	0	0	0	0	0	0	0	0	0	0	0	0	0	0	0	0	0	0	0	0	0	0	0	0	0	0	0	0	0

[18F]FMZ Automated Production Recipe Continued

V47 (0=off, 1=on)	V48 (0=off, 1=on)	V49 (0=off, 1=on)	V50 (0=off, 1=on)	HPLC V1 (0=inject, 1=load)	HPLC V2 (0=inject, 1=load)	HPLC V3/4 (1-4 position)	Reactor 1 Temp °C (0-255)	Reactor 2 Temp °C (0-255)	Reactor 1 Tube (0=up, 1=down)	Reactor 2 Tube (0=up, 1=down)	Reactor 1 Stirrer (0=off, 1=on)	Reactor 2 Stirrer (0=off, 1=on)	Flask 1 Stirrer (0=off, 1=on)	Flask 2 Stirrer (0=off, 1=on)	Vacuum Pump (0=off, 1=on)	HPLC Pump Flow (0.0-50.0 mL/min)	HPLC Eluent A% (0-100)	HPLC Eluent B% (0-100)	HPLC Eluent C% (0-100)	HPLC Eluent D% (0-100)	Gradient Time (xx.xx min)	Inter. Vial 1 Temp °C (0-150)	Inter. Vial 2 Temp °C (0-150)	Inter. Vial 1&2 Stirrers (0=off, 1=on)	Inter. Vial 1 V51 (0=off, 1=on)	Inter. Vial 2 V52 (0=off, 1=on)
0	0	0	0	0	0	1	0	0	0	0	0	0	0	0	0	4.0	90	10	0	0	0.00	0	0	0	0	0
0	0	0	0	0	0	1	0	0	0	0	0	0	0	0	0	4.0	90	10	0	0	0.00	0	0	0	0	0
0	0	0	0	0	0	1	0	0	0	0	0	0	0	0	0	4.0	90	10	0	0	0.00	0	0	0	0	0
0	0	0	0	0	0	1	0	0	0	0	0	0	0	0	0	4.0	90	10	0	0	0.00	0	0	0	0	0
0	0	0	0	0	0	1	0	0	0	0	0	0	0	0	0	4.0	90	10	0	0	0.00	0	0	0	0	0
0	0	0	0	0	0	1	0	0	0	0	0	0	0	0	0	4.0	90	10	0	0	0.00	0	0	0	0	0
0	0	0	0	0	0	1	0	0	0	0	0	0	0	0	0	4.0	90	10	0	0	0.00	0	0	0	0	0
0	0	1	0	0	0	1	0	0	0	0	0	0	0	0	0	4.0	90	10	0	0	0.00	0	0	0	0	0
0	0	0	0	0	0	1	0	0	0	0	0	0	0	0	0	4.0	90	10	0	0	0.00	0	0	0	0	0
0	0	0	0	0	0	1	0	0	0	0	0	0	0	0	0	4.0	90	10	0	0	0.00	0	0	0	0	0
0	0	0	0	0	0	1	0	0	0	0	0	0	0	0	0	4.0	90	10	0	0	0.00	0	0	0	0	0
0	0	0	0	0	0	1	0	0	0	0	0	0	0	0	0	4.0	90	10	0	0	0.00	0	0	0	0	0
0	0	0	0	0	0	1	0	0	0	0	0	0	0	0	0	4.0	90	10	0	0	0.00	0	0	0	0	0
0	0	0	0	0	0	1	0	0	0	0	0	0	0	0	0	4.0	90	10	0	0	0.00	0	0	0	0	0
0	0	0	0	0	0	1	0	0	0	0	0	0	0	0	0	4.0	90	10	0	0	0.00	0	0	0	0	0
0	0	1	0	0	0	1	0	0	0	0	0	0	0	0	0	4.0	90	10	0	0	0.00	0	0	0	0	0
0	0	0	0	0	0	1	0	0	0	0	0	0	0	0	0	4.0	90	10	0	0	0.00	0	0	0	0	0
0	0	0	0	0	0	1	0	0	0	0	0	0	0	0	0	4.0	90	10	0	0	0.00	0	0	0	0	0
0	0	0	0	0	0	1	0	0	0	0	0	0	0	0	0	4.0	90	10	0	0	0.00	0	0	0	0	0
0	0	0	0	0	0	1	0	0	0	0	0	0	0	0	1	4.0	90	10	0	0	0.00	0	0	0	0	0
0	0	0	1	0	0	1	60	0	0	0	0	0	0	0	1	4.0	90	10	0	0	0.00	0	0	0	0	0
0	0	0	1	0	0	1	60	0	0	0	0	0	0	0	1	4.0	90	10	0	0	0.00	0	0	0	0	0
0	0	0	1	0	0	1	85	0	0	0	1	0	0	0	0	4.0	90	10	0	0	0.00					
0	0	0	1	0	0	1	85	0	0	0	1	0	0	0	1	4.0	90	10	0	0	0.00	0	0	0	0	0
0	0	0	1	0	0	1	85	0	0	0	1	0	0	0	1	4.0	90	10	0	0	0.00	0	0	0	0	0
0	0	0	1	0	0	1	60	0	0	0	1	0	0	0	0	4.0	90	10	0	0	0.00	0	0	0	0	0
0	0	0	1	0	0	1	55	0	0	0	1	0	0	0	0	4.0	90	10	0	0	0.00	0	0	0	0	0
0	0	0	0	0	0	1	120	0	0	0	1	0	0	0	0	4.0	90	10	0	0	0.00	0	0	0	0	0
0	0	0	1	0	0	1	45	0	0	0	1	0	0	0	0	4.0	90	10	0	0	0.00	0	0	0	0	0
0	0	0	1	0	0	1	0	0	1	0	0	0	0	0	0	4.0	90	10	0	0	0.00	0	0	0	0	0
0	0	0	1	0	0	1	45	0	0	0	1	0	0	0	0	4.0	90	10	0	0	0.00	0	0	0	0	0
0	0	1	0	0	0	1	45	0	0	0	1	0	0	0	0	4.0	90	10	0	0	0.00	0	0	0	0	0
0	0	0	1	0	0	1	45	0	1	0	0	0	0	0	0	4.0	90	10	0	0	0.00	0	0	0	0	0
0	0	0	1	0	0	1	45	0	0	0	0	0	0	0	0	4.0	90	10	0	0	0.00	0	0	0	1	0
0	0	0	1	0	0	1	45	0	0	0	0	0	0	0	0	4.0	90	10	0	0	0.00	0	0	0	1	0
0	0	0	0	1	0	1	45	0	0	0	0	0	0	0	0	5.0	63	37	0	0	40.00	0	0	0	0	0
0	0	0	0	1	0	1	45	0	0	0	0	0	1	0	0	5.0	0	0	80	20	0.00	0	0	0	0	0
0	0	1	0	1	0	1	45	0	0	0	0	0	0	0	0	5.0	0	0	80	20	0.00	0	0	0	0	0
0	0	0	1	1	0	1	45	0	0	0	0	0	0	0	0	5.0	0	0	80	20	0.00	0	0	0	0	0
0	0	0	1	1	0	1	45	0	0	0	0	0	0	0	0	5.0	0	0	80	20	0.00	0	0	0	0	0
0	0	0	1	1	0	1	45	0	0	0	0	0	0	0	0	5.0	0	0	80	20	0.00	0	0	0	0	0
0	0	1	0	0	0	1	0	0	0	0	0	0	0	0	0	5.0	0	0	80	20	0.00	0	0	0	0	0
0	1	0	1	0	0	1	0	0	0	0	0	0	0	0	0	5.0	0	0	80	20	0.00	0	0	0	0	0
0	0	0	0	0	0	1	0	0	0	0	0	0	0	0	0	0.0	100	0	0	0	0.00	0	0	0	0	0
0	0	0	0	0	0	1	0	0	0	0	0	0	0	0	0	0.0	0	0	0	0	0.00	0	0	0	0	0
